# Complete Genome Sequence of Streptococcus mutans Strain MD, Which Produces Highly Potent Mutacins

**DOI:** 10.1128/MRA.00616-20

**Published:** 2020-08-13

**Authors:** Saswati Biswas, Indranil Biswas

**Affiliations:** a Department of Microbiology, Molecular Genetics, and Immunology, University of Kansas Medical Center, Kansas City, Kansas, USA; University of Maryland School of Medicine

## Abstract

Here, we report the complete genome sequence of Streptococcus mutans strain MD, which produces potent mutacins capable of inhibiting streptococci. MD is a relatively uncharacterized strain whose genome information was unavailable. This study provides useful information for comparative genomic study and for understanding the repertoire of mutacins in S. mutans.

## ANNOUNCEMENT

Streptococcus mutans is one of the principal etiological agents of human dental caries (1). The human oral microbiome is composed of >700 different bacterial species, and >30% of them belong to the genus *Streptococcus* (2, 3). S. mutans produces numerous small antibacterial peptide bacteriocins, called mutacins; they inhibit the growth of competing bacteria, including streptococci. This suppression of other bacteria allows S. mutans to become one of the dominant species in the oral cavity to form dental caries. Bacteriocins are broadly classified into two classes, namely, lantibiotics (class I), which are peptides containing dehydrated amino acids and unusual amino acids such as lanthionine, and nonlantibiotics (class II), which are linear unchanged peptides. The nature and potency of mutacins produced by S. mutans are highly strain dependent and vary significantly (4–6).

Our group has been working with a prolific mutacin-producing strain that was previously referred to as T8 (7) (M. Duncan, personal communication). However, this T8 strain is not the same as the original T8 strain that produces mutacin II (8). We also observed that this particular isolate displayed a very different inhibitory spectrum and produced a two-peptide lantibiotic, Smb, similar to that produced by the GS-5 strain (7, 9, 10); however, its inhibitory potency is greater than that of the Smb produced by the GS-5 strain. Therefore, we renamed this isolate MD to distinguish it from the original T8 strain (8). To study this isolate further, we determined its complete genome sequence.

The bacterial culture was cultivated at 37°C in Todd-Hewitt medium (BBL) supplemented with 0.2% yeast extract, under microaerophilic conditions. Genomic DNA was isolated using a MasterPure DNA purification kit (Lucigen) as described previously (11–13). The quality and quantity of the isolated genomic DNA were verified by using gel electrophoresis and a NanoDrop spectrophotometer (Thermo Fisher Scientific), respectively. DNA was sheared using a g-TUBE (Covaris), and SMRTbell DNA libraries were prepared using the Express template preparation kit v2.0 (Pacific Biosciences) according to the manufacturer’s protocol. Samples were pooled into a single multiplexed library, size selected using BluePippin (Sage Sciences), and sequenced on a Sequel II system with Sequel II chemistry v1.0, at SNPsaurus. Raw reads were converted to the fasta format using SAMtools (14) and were quality controlled using FastQC v0.10.1 with default parameters. Canu v1.7 (15) was used with default settings to assemble the genome from 29,320 raw reads (*N*_50_ value of 9,099 bp) with a total of 221,612,731 bases. The assembled genome had 113-fold genome coverage. The genome harbors a circular chromosome of 2,009,428 bp (GC content of 36.89%) and a plasmid of 13,108 bp (GC content of 43%) with homology to pVA838 (16). The genome annotation was carried out using the IGS Prokaryotic Annotation Pipeline at the Institute for Genome Sciences at the University of Maryland (17) and set *oriC/dnaA* as the start.

Genome analysis by CRISPRCasFinder (18) suggests that the MD genome harbors three CRISPR/Cas loci, which is similar to the S. mutans GS-5 strain. We analyzed the genome to predict putative biosynthetic gene clusters (BGCs), including those for bacteriocins (mutacin), using the antiSMASH (19) and BAGEL4 (20) servers with default search options. As expected, the MD genome contains genes for the lantibiotic Smb (7, 10) and several nonlantibiotics, as well as several BGC loci. The genome sequencing also identified three new methylation motifs in S. mutans in addition to the GATC motif. The new motifs are ATGCAT (m6A), CNGCGNCGGNTNNNG, and CTNNNNTATANCNNNAC. This study will add valuable information regarding the evolution and production of the lantibiotic Smb among various S. mutans isolates.

### Data availability.

The complete genome sequence of the S. mutans MD strain has been deposited in GenBank under the accession numbers CP044493 (chromosome) and CP044494 (plasmid). The GenBank assembly number for the genome is GCA_008831325.1. Raw reads have been deposited in the SRA under the accession number SRR11818585.
